# Em Busca da Qualidade de Imagem Ideal na Angiotomografia Cardíaca Pediátrica

**DOI:** 10.36660/abc.20201279

**Published:** 2021-01-27

**Authors:** Daniel Faria, João B. Augusto

**Affiliations:** 1 Serviço de Cardiologia Hospital Prof. Doutor Fernando Fonseca Amadora Portugal Serviço de Cardiologia, Hospital Prof. Doutor Fernando Fonseca, Amadora - Portugal; 2 Institute of Cardiovascular Sciences University College London Londres Reino Unido Institute of Cardiovascular Sciences, University College London, Londres – Reino Unido; 3 Advanced Cardiac Imaging Department Barts Heart Centre Londres Reino Unido Advanced Cardiac Imaging Department, Barts Heart Centre, Londres – Reino Unido

**Keywords:** Cardiopatias Congênitas, Diagnóstico por Imagem, Adrenérgicos, Metropolol, Frequência Cardíaca, Angiotomografia

“Não existe revelação mais nítida da alma de uma sociedade do que a forma como esta trata as suas crianças”. Anos após a sua morte, essas palavras prolíficas de Nelson Mandela ainda ressoam universalmente com nossos fundamentos morais e éticos e, como pesquisadores, estamos muito felizes em saber que a ciência caminha no caminho certo da história.

Existem vários desafios metodológicos e éticos na realização de pesquisas em crianças. No entanto, não pode haver progresso no atendimento clínico pediátrico sem pesquisas nesta população, cujos achados também podem ser relevantes para a medicina de adultos. Dado que aproximadamente 1% das crianças nascidas terão algum tipo de doença cardíaca significativa,^1^ é de fundamental importância maximizar o perfil de segurança e eficácia das intervenções diagnósticas e terapêuticas. A angiotomografia cardíaca (ATC) está sendo cada vez mais utilizada, mas sua precisão diagnóstica em crianças é altamente dependente da qualidade ideal da imagem, minimizando a exposição à radiação tanto quanto possível.

Mesmo com equipamentos modernos, a qualidade da imagem da angiotomografia cardíaca (ATC) ainda é altamente dependente de uma frequência cardíaca (FC) estável e relativamente lenta.^2^ Para atingir as condições ideais pré-exame, geralmente se preconiza a administração de betabloqueadores e as sociedades de cardiologia já publicaram diversos documentos que fornecem orientação para a seleção e administração de pacientes.^3 , 4^ No entanto, o diferente comportamento farmacocinético dos betabloqueadores em pacientes pediátricos (além da FC basal mais alta, movimento corporal e artérias coronárias menores) lançam uma sombra em relação à estratégia e dosagem ideais para obter imagens de alta qualidade sem incorrer no risco de bradiarritmias.^5^ Os betabloqueadores devem ser administrados em dose apropriada, dados os possíveis efeitos colaterais, mas as doses e os protocolos geralmente variam entre os serviços de saúde.

De Oliveira Nunes et al.,^6^ conduziram um interessante estudo que lança uma luz muito necessária e esperada sobre essa incerteza. O objetivo do estudo foi esclarecer a segurança e eficácia de um protocolo de metoprolol em uma série de pacientes pediátricos ambulatoriais encaminhados para ATC. Resumimos o protocolo usado na Figura 1 . Resumidamente, se a FC de um paciente estiver abaixo de 60 bpm, nenhuma redução da FC será necessária. Para aqueles com FC de pelo menos 60 bpm, na ausência de contraindicações ao uso de betabloqueador (por exemplo, estenose aórtica grave ou hipertensão pulmonar significativa), utilizou-se protocolo com metoprolol ( Figura 1 ). A qualidade média da imagem resultante foi próxima do ideal na maioria dos casos, com apenas 14% efetivamente abaixo do ideal, conforme considerado pelos pesquisadores. Os autores devem ser elogiados pelas adaptações ao protocolo, que vieram com o tempo e a experiência. Eles começaram usando o tratamento de primeira linha com metoprolol oral seguido por metoprolol IV se a FC estivesse persistentemente elevada (acima de 70 bpm). No entanto, eles observaram que não houve redução adicional significativa na FC com metoprolol IV, então interromperam seu uso a partir de 2013. Embora alguns protocolos ainda defendam seu uso na prática clínica, principalmente em adultos,^4^ isso exige estudos prospectivos dedicados para responder a essa questão, principalmente em crianças de alto risco, nas quais o uso de betabloqueadores intravenosos pode aumentar o risco com pouco benefício.

**Figura 1:**
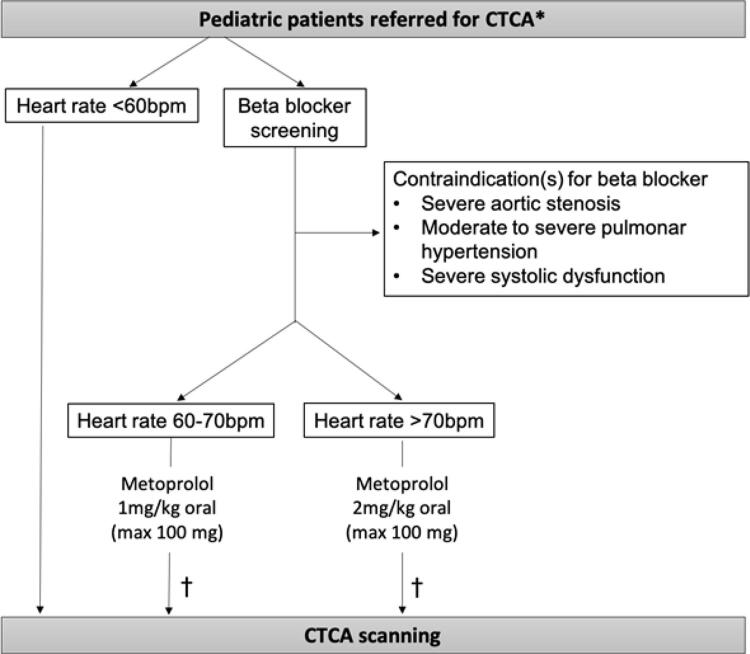
– Resumo do protocolo de estudo utilizado por De Oliveira Nunes e cols.6 ATC: angiotomografia cardíaca. *Pacientes entre 6 e 18 anos. † Os pesquisadores pararam de dar uma dose IV adicional de metoprolol (de 2013 em diante) no caso de frequência cardíaca persistente >70 bpm uma hora após a dose oral, pois nenhuma redução adicional significativa da frequência cardíaca foi observada.

A tecnologia da tomografia computadorizada está em constante evolução e permite melhor qualidade de imagem, com o aumento da largura do detector, tempos de aquisição de imagem mais curtos e *gating* ideal no ECG. Uma característica particularmente interessante desse estudo é o amplo período que abrangeu, de 2007 a 2016. Isso significa que ocorreram avanços tecnológicos: foram utilizados equipamentos de diferentes gerações e tubos com diferentes potências, portanto mais representativos do mundo real.

Algumas lacunas nas evidências, entretanto, ainda existem. Diferentes betabloqueadores podem ser usados (com possíveis diferenças na eficácia), pacientes com menos de 6 anos não foram incluídos neste estudo (que são mais propensos a contribuir para pior qualidade de imagem) e um desenho de estudo prospectivo com um grupo de controle aumentaria o nível de evidência. Pesquisas adicionais nessas áreas são, portanto, desejáveis.

Mesmo as modalidades de ponta podem ter dificuldades com a obtenção adequada de imagens cardíacas ideais, particularmente na população pediátrica. Portanto, é importante focar nos pontos que ainda podem ser melhorados em nossa prática clínica, especificamente na FC. Esse aspecto deve ser bem estabelecido entre as equipes de cardiologistas pediátricos e técnicos de radiologia, antes de se tentar esquemas de aquisição (possivelmente desnecessários) que poderiam resultar em maior exposição à radiação ou mesmo exigir sedação. Soluções inteligentes como as apresentadas nos Arquivos Brasileiros de Cardiologia por De Oliveira Nunes e cols.6 são sempre bem-vindas e nos permitem subsidiar a tomada de decisões em nossa prática clínica.

## References

[1] . Hoffman JL, Kaplan S. The incidence of congenital heart disease. J Am Coll Cardiol. 2002;39(12):1890-900. 10.1016/s0735-1097(02)01886-7 12084585

[2] . Sun G, Li M, Jiang XS, Li L, Peng ZH, Li G-Y, et al. 320-detector row CT coronary angiography: effects of heart rate and heart rate variability on image quality, diagnostic accuracy and radiation exposure. Br J Radiol. 2012;85(1016):e388-e394. 10.1259/bjr/92160185 PMC3587067 22374285

[3] . Abbara S, Blanke P, Maroules CD, Cheezum M, Choi AD, Han BK, et al. SCCT guidelines for the performance and acquisition of coronary computed tomographic angiography: A report of the society of Cardiovascular Computed Tomography Guidelines Committee: Endorsed by the North American Society for Cardiovascular Imaging (NASCI). J Cardiovasc Comput Tomogr. 2016 Nov-Dec;10(6):435-49. 10.1016/j.jcct.2016.10.002 27780758

[4] . Royal College of Physicians, Royal College of Radiologists and the British Society of Cardiovascular Imaging. Standards of practice of computed tomography coronary angiography (CTCA) in adult patients. 2014. [Internet] [Cited in 2020 Oct 20] Available from:rcr.ac.uh/system/files/publication/files/BFCR14%2816%29_CTCA.pdf

[5] . Watanabe H, Kamiyama H, Kato M, Komori A, Abe Y, Ayusawa M. Appropriate use of a beta-blocker in paediatric coronary CT angiography. Cardiol Young. 2018 Oct;28(10):1148-53. 10.1017/S104795111800118X 30079850

[6] . De Oliveira Nunes M, Witt DR, Casey SA, Stanberry LI, Caye DJ, Chu BJ, et al. Segurança, eficácia e protocolo de dose de metropolol para redução de frequência cardíaca em Pacientes pediátricos externos que passaram por angiografia cardíaca por TC. Arq Bras Cardiol. 2021; 116(1):100-105. 10.36660/abc.20190892 PMC8159495 33566972

[7] . Young C, Taylor AM, Owens CM. Paediatric cardiac computed tomography: a review of imaging techniques and radiation dose consideration. Eur Radiol. 2011 Mar;21(3):518-29. 10.1007/s00330-010-2036-8 21188593

