# Supplementary material for: Em Busca da Qualidade de Imagem Ideal na Angiotomografia Cardíaca Pediátrica
Source: Arq Bras Cardiol. 2021 Jan 27;116(1):106–7. [Article in Portuguese] doi: 10.36660/abc.20201279 (PMC8159505; doi:10.36660/abc.20201279)
Supplement: Translation

# Translation: In Search for Optimal Image Quality in Pediatric Cardiac CT Angiogram

**Authors:** Daniel Faria, João B. Augusto

**Affiliations:** 1 Portugal Serviço de Cardiologia, Hospital Prof. Doutor Fernando Fonseca, Amadora - Portugal; 2 Reino Unido Institute of Cardiovascular Sciences, University College London, Londres – Reino Unido; 3 Reino Unido Advanced Cardiac Imaging Department, Barts Heart Centre, Londres – Reino Unido

**Keywords:** Heart Defects, Congenital, Diagnostic Imaging, Adrenergic Agents, Metropolol, Heart Rate, Angiotomography

“There can be no keener revelation of a society’s soul than the way in which it treats its children”. Years after his death, these Nelson Mandela’s prolific words still resonate universally with our moral and ethical foundations and, as researchers ourselves, we are very happy to know that Science walks on the right path of history.

There are a number of methodological and ethical challenges of performing research in children. However, there can be no progress in pediatric clinical care without research in this population, whose findings may also otherwise be relevant to adult medicine. Given that approximately 1% of born children will have some kind of significant heart disease,^1^it is of crucial importance to maximize the safety and efficacy profile of diagnostic and therapeutic interventions. Computed tomography cardiac angiography (CTCA) is being increasingly used, but its diagnostic accuracy in children is highly dependent on optimal image quality while minimizing radiation exposure as much as possible.

Even with modern scanners, image quality in CTCA is still highly dependent on a stable and relatively slow heart rate (HR).^2^To achieve optimal pre-scan conditions, beta-blocker administration is often advocated and a number of societal documents have been published providing guidance for patient selection and administration.^3 , 4^Nevertheless, the different pharmacokinetic behavior of beta-blockers in pediatric patients (in addition to the higher baseline HR, body movement and smaller coronary arteries) cast a shadow regarding the optimal strategy and dosage to obtain high quality images without incurring in the risk of bradyarrhythmias.^5^Beta-blockers should be given at an appropriate dose given the potential side effects, but doses and protocols typically vary among facilities.

De Oliveira Nunes et al.^6^share an elegant study that sheds a much needed and awaited light on this uncertainty. The aim of this study was to clarify the safety and efficacy of a metoprolol protocol in a series of pediatric outpatients referred for CTCA. We have summarized the protocol used in the Figure 1 . Briefly, if a patient’s HR is below 60bpm, then no HR reduction is necessary. For those with a HR of at least 60bpm, in the absence of contraindications to beta-blocker use (e.g. severe aortic stenosis or significant pulmonary hypertension), a protocol using metoprolol was employed ( Figure 1 ). The average image quality that resulted was close to optimal in most cases, with only 14% effectively being suboptimal as deemed by the researchers. The authors should be commended for the protocol adaptations that came with time and experience. They first started by treating patients with oral with oral metoprolol followed by IV metoprolol if the HR was persistently elevated (above 70bpm). However, they elegantly noted that there was no significant additional reduction in HR with IV metoprolol, so they stopped its use from 2013 onwards. Although some protocols still advocate its use in clinical practice, particularly in adults,^4^this calls for dedicated prospective studies to answer this question, particularly in high-risk children, in whom the use of IV beta-blockers can increase risk with little benefit.

**Figure 1:** – Summary of the study protocol used by De Oliveira Nunes et al.6 CTCA: computed tomography cardiac angiography. * Patients between 6 and 18 years old. † The investigators stopped giving an additional IV dose of metoprolol (from 2013 onwards) in the case of persistent heart rate >70bpm one hour after oral dose as no significant additional heart rate reduction was appreciated.

CT technology is continuously evolving and allows better image quality, with increasing detector width, shorter CT acquisition times and optimal ECG-gating. One particularly interesting feature of this study is the wide period of time it encompassed, from 2007 to 2016. This means that technological advances took place: different generation scanners and different tube potentials were used, thus more likely representative of the real world.

Some gaps in evidence, however, still exist. Different beta-blockers can be used (with potential differences in efficacy), patients younger than 6 years were not included in this study (who are more likely to contribute to poorer image quality) and a prospective study design with a control group would increase the evidence level. Additional investigation in these areas are thus desirable.

Even high-end modalities can struggle with appropriate attainment of optimal cardiac images, particularly in the pediatric population. It is thus important to focus on the points that can still be improved in our clinical practice, and here specifically on HR. This aspect should be well established among teams of pediatric cardiologists and radiology technicians, before attempting (potentially unnecessary) acquisition schemes that could result in more radiation exposure or even require sedation. Elegant solutions such as the ones presented in *Arquivos Brasileiros de Cardiologia* by De Oliveira Nunes et al.^6^are always welcome and allow us to support decision-making in our clinical practice.
